# Impact of cardiopulmonary resuscitation duration on the neurological outcomes of out-of-hospital cardiac arrest

**DOI:** 10.1186/s12245-022-00418-4

**Published:** 2022-03-19

**Authors:** Hissah Albinali, Arwa Alumran, Saja Alrayes

**Affiliations:** 1 Royal Commission Hospital, P.O.Box 11994, Jubail Industrial City, 31961 Saudi Arabia; 2 Health Information Management and Technology Department, College of Public Health, Imam Abdulrahman bin Faisal University, Dammam, Saudi Arabia

**Keywords:** Cardiac arrest, Patient outcomes, Cardiopulmonary resuscitation, Neurological outcome, Cerebral performance category

## Abstract

**Background:**

Patients experiencing cardiac arrest outside medical facilities are at greater risk of death and might have negative neurological outcomes. Cardiopulmonary resuscitation duration affects neurological outcomes of such patients, which suggests that duration of CPR may be vital to patient outcomes.

**Objectives:**

The study aims to evaluate the impact of cardiopulmonary resuscitation duration on neurological outcome of patients who have suffered out-of-hospital cardiac arrest.

**Methods:**

This is a quantitate cross-sectional study where data is collected from emergency cases handled by a secondary hospital in industrial Jubail, Saudi Arabia, between January 2015 and December 2020. There were 257 out-of-hospital cardiac arrest cases, 236 of which resulted in death. The outcome is the survival of OHCA or death, and the neurological outcome by the cerebral performance category (CPC) score for survivors. A score of 1 or 2 defined as good CPC outcome and 3, 4, and 5 as poor outcome.

**Results:**

The mean for the duration of emergency CPR procedures in surviving patients is 26.5 ± 7.20 min, whereas in patients who died after the procedure it is 29.6 ± 9.15 min. Bivariate analysis showed no significant association between duration of CPR and Cerebral Performance Category (CPC) outcome but could be significant if the sample size is large. Age, however, is significantly related to the survivorship of OHCA and to a better CPC outcome. Younger patients are more likely to have better CPC outcome. A good CPC outcome was reported with a limited duration of 8.1 min of CPR, whereas, poor CPC outcomes were associated with prolonged periods of CPR, 13.2 min.

**Conclusion:**

Cardiopulmonary Resuscitation Duration out-of-hospital cardiac arrest does not significantly influence the patient neurological outcome in the current study hospital. Variables such as the patient population's uniqueness, underlying medical conditions, or the specific study conditions may explain this variance between the bivariate analysis and the study conclusion. Therefore, a more comprehensive study is recommended in future.

## Introduction

Out-of-hospital cardiac arrest (OHCA) is a major public health concern across the world. Statistics demonstrate that an average of 330,000 people in the USA and 275,000 people in Europe experience OHCAs every year [1]. Moreover, the same statistics demonstrate that the survival rates for OHCAs are low. Cardiac arrests occur when the heart has stopped pumping blood to the body, accounting for many deaths across the world; most cardiac arrests happen outside hospital facilities [2]. According to Paratz et al. [3], cardiac arrests account for 20% of all Western deaths. Cardiac arrest is the result of a number of factors, and several scholars have attempted to determine the relationship between various prehospital factors such as the first recorded rhythm, age, and the survival rate after cardiac arrests. OHCAs affect society negatively and in a significant way [4]. For instance, patients’ family members experience emotional burdens and the medical staff suffers vital implications. To rescue a cardiac arrest patient, medical professionals perform CPR, which includes rescue breathing such as mouth-to-mouth resuscitation and chest compressions [5].

Patients experiencing cardiac arrest might have negative neurological outcomes. The outcomes of cardiac arrest are mainly related to its effects on the neurological system. The neurological outcome can be good or poor, depending on a variety of factors. Therefore, it is important to perform this assessment in situations where CPR procedures have been administered.

Park et al. [6] found that of 65 cardiac arrest patients investigated, only 24.6% had positive neurological outcomes, whereas 75.4% had poor outcomes. Most poor prognoses are related to the withdrawal of life-sustaining therapy after the cardiac arrest. Therefore, it is important to carry out a neurological examination after cardiac arrest, including an examination of the different reflexes and motor responses. This examination can prompt interventions that are important to prevent further complications to the patient.

Duration plays a significant and crucial role in the identification of neurological outcomes after cardiac arrest. Health care workers should record good or poor neurological outcomes after a patient’s cardiac arrest. A study by Yukawa et al. [7] indicated that patients had a good neurological outcome when the CPR duration was between 40 min. It also is important to initiate CPR within 40 min of the onset of cardiac arrest, either prehospital or in the hospital. With a shorter duration of CPR, the outcomes are positive.

The above mentioned literature provides evidence of the importance of understanding the overall effects of CPR duration on neurological outcomes. Health care professionals have made attempts to terminate CPR’s use to stop the effects that can arise from its administration. Nonetheless, various organizations have argued that the procedure should not be discontinued entirely; instead, health care professionals should be granted the right to weigh the benefits of its administration as part of the CPR protocol [1]. No study has been conducted in Industrial Jubail, Saudi Arabia, to determine the impact of CPR duration on neurological outcomes of OHCA and the influencing factors. This study sought to explore the relationship between CPR duration and the achievement of neurologically favorable outcomes at a secondary hospital in Industrial Jubail.

## Research methodology

### Research design

This is a quantitative cross-sectional design to evaluate the impact of CPR duration on neurological outcomes of Out of Hospital Cardiac Arrest (OHCA).

### Study setting

This research was carried out at a major hospital in Industrial Jubail, Eastern Saudi Arabia. The study institution has eight ambulances and offers care for various groups of patients who are critically ill and require comprehensive stabilization. About two to three prehospital cardiac arrest incidents occur each month; the attending Emergency Medical Service (EMS) specialist will request assistance and initiate efforts aimed at resuscitating the patient in collaboration with other members of the team. According to AHA guidelines, when providing CPR, the EMS specialist in charge is required to fill out a CPR form and ensure that it is duly completed after the event.

### Study participants

This study included only the patients’ OHCA data, which was extracted from a hospital database. Both pediatric and adult patients of both genders were included.

### Variables

The dependent variables assessed in this study included patients’ neurological outcomes, which were measured using the Cerebral Performance Category (CPC) scale [8]. The outcome variable was categorized according to either good or poor neurological outcomes based on previous studies [9].

The independent variables assessed in this study included patients’ gender, age, and the duration of CPR measured in minutes until the return of spontaneous circulation was achieved in prehospital settings or after hospital admission.

### Data collection

The hospital’s electronic medical database was utilized retrospectively to collect the participating patients’ data; this was important because it provided easy access to important demographic data and patient outcome information. The cardiac arrest cases sampled in this study were reported between 2015 and 2020.

### Instruments

The cerebral performance category (CPC) scale was used to assess neurological outcomes from resuscitation attempts at hospital discharge. According to numerous studies [8], a CPC score of 1 or 2 shows a good neurological outcome (favorable outcome), whereas a score of 3, 4, or 5 shows a poor neurological outcome (unfavorable outcome, often severe neurological outcome, or death).

### Procedure and timeline

The cerebral performance category (CPC) assessment of patients’ neurological outcomes was done at hospital discharge. Data were gathered during a 2-month period between January and February 2021.

### Ethics and limitations

Ethical approval was obtained from the Institutional Review Board at Imam Abdulrahman Bin Faisal University, Dammam, Saudi Arabia (IRB-PGS-2021-03-048) and the Institutional Review Board at Royal Commission Hospital, Industrial Jubail, Saudi Arabia (IRB-RCH-013). The medical chart research was carried out confidentially without the exposure of any of the participants’ personal information.

### Analysis

To determine and illustrate a descriptive summary of the findings, means and standard deviations were calculated for continuous variables, Counts and frequencies were used for categorical variables. Additionally, bivariate analysis was carried out to evaluate the association between the cerebral performance category (CPC) results indicating neurological outcomes and patient’s ender using *χ*^2^ test, and between the effect of CPR duration and the patients age on the neurological outcome were assessed using Mann-Whitney *U* test. IBM SPSS Statistics (Version 25) (IBM [10]) was utilized to carry out the analysis.

## Results

Of the 257 patients included in this study, 184 (71.6%) were males and 73 (28.4%) were females. The mean and standard deviation of the patients’ ages were 48.4 ± 23.2 years (age range: 7–94 years). The overall mean and standard deviation of the duration of CPR were found to be 29.3 ± 9 min (with an actual range of 9–60 min). Almost half of the patients in the study had an OHCA because of a medical condition (*n*= 144, 56%), while the rest was caused by trauma (*n* = 113, 44%). The patients’ characteristics are summarized in Table 1.

**Table 1:** Demographic characteristics of study participants

Variable	Frequency (%) *n* = 257
**Gender**	
Male	184 (72)
Female	73 (28)
**Cause of OHCA**	
Medical	144 (56)
Trauma	113 (44)
**Age (years)**	48.4 ± 23.2 (range 7–94)
**Duration of CPR (min)**	29.3 ± 9 (range 9–60)

Bivariate analysis using *χ*2 test to assess the association between patients’ neurological outcomes and their gender. It shows that half of the female survivors had good cerebral performance category (CPC) outcome (*n* = 2, 50%), while only 41% of male’s survivors (*n* = 7) had good outcome. Nevertheless, there was no statistically significant difference in cerebral performance category (CPC) outcomes between gender groups (*χ*2 = 1.75, *p* = 0.748) (Table 2).

**Table 2:** Association between demographic factors, cause of death, and duration of CPR with CPC

	CPC^**a**^ outcome		***χ***^**2**^ (***P*** value)
	Good	Poor	
	***n*** (%)	***n*** (%)	
**Gender**			
Female	2 (50%)	2 (50%)	.175 (0.748)
Male	7 (41.2%)	10 (58.8%)	
**Cause of death**			
Medical	2 (22.2%)	7 (77.8%)	2.738 (0.098)
Trauma	7 (58.3%)	5 (41.7%)	
	**Mean rank**	**Mean rank**	**Mann-Whitney** ***U*** **test (*P*** **value)**
**Duration of CPR (min)**	8.1	13.2	27.500 (0.059)
**Age (years)**	5.8	14.9	7.000 (0.001)

^a^*CPC* Cerebral Performance Category scale

In addition, the assessment was done on the cause of the OHCA, i.e., either medical or traumatic causes. The cerebral performance category (CPC) outcomes in patients who developed an OHCA due to medical causes showed that two (22.2%) individuals had good CPC outcomes and seven (77.8%) recorded poor CPC outcomes. The CPC outcomes were a bit better in patients who developed OHCA due to traumatic causes, more than half had good CPC outcomes (*n* = 7, 58.3%). However, there was no statistically significant difference in CPC outcomes between the two causes of OHCA (medical or trauma; *p* = 0.098).

Furthermore, Mann-Whitney *U* test using to assess the neurological outcomes and duration of CPR. Regarding the duration of CPR in minutes, a good CPC outcome was reported with a (mean) limited duration of 8.1 min of CPR; whereas, poor CPC outcomes were associated with prolonged periods of CPR, 13.2 min (mean). Similarly, youthfulness was associated with good CPC outcomes as revealed by the mean age of 24 years, whereas a mean age of 48 years was aligned with a poor CPC outcome; this difference between age groups was statistically significant (Mann-Whitney *U* = 7.000, *P* = 0.001). Therefore, a low sample size of survivors as a reason of the bivariate analysis was not statistically significant, but could be significant if our study had a larger sample.

## Discussion

Saudi Arabia’s population is unique compared to other regions. The CPRs knowledge in the country among non-medical professionals is significantly insufficient. One study indicated that 60% of Saudis do not know what CPR is. Only 5% of the population have had formal CPR training, 5% attended various campaigns, while 40% learned CPR skills from the media. Saudis with higher education demonstrated a higher level of knowledge of CPR compared to the less educated. At the same time, insufficient knowledge is the main obstacle for individuals who had acquired CPR skills via the media. These factors may adversely affect Saudi Arabians’ capacity to respond in the instance of cardiac arrest. The government of Saudi Arabia has prioritized improving medical care services at every level, including primary, secondary, and tertiary. As a result, the population’s medical condition has substantially improved in the past several decades. Nonetheless, a raft of challenges causes problems to the health care framework, particularly in the secondary hospitals. Such hospitals have a shortage of qualified health professionals, inadequate monetary resources, high demand due to free accessibility to the general population, and lack of proper expertise and equipment to counter the fluctuating disease patterns. Because cardiac arrest continues to cause a significant number of deaths in Saudi Arabia, it is important to investigate the effectiveness of CPR procedures in saving the lives of affected individuals. To achieve that goal, this study on the effects of CPR duration on neurological outcomes, together with the existing literature, offers insights concerning the termination of the medical procedure. This study aimed to investigate the impact of CPR duration on neurological outcomes to demonstrate those outcomes in cardiac arrest patients. This was achieved through the utilization of a qualitative cross-sectional design to assess the impact of CPR duration on neurological outcomes. The dependent variables in the study included good or poor neurological outcomes that were assessed against independent variables such as age, sex, and duration of CPR. This study inferred that shorter CPR durations led to favorable neurological outcomes and survival and study reported a strong association between CPC scores and rapid recovery trends. A good outcome was reported with a limited duration of 8.1 min of CPR administration, whereas poor CPC outcomes were associated with prolonged periods of CPR with an average of 13.2 min. These results correspond with those from a study by Xue et al. [9], who established that shorter CPR durations result in higher chances of survival compared to increased CPR durations. This is also in agreement with a study by Scott et al. [11], who established that individuals who were exposed to shorter CPR durations had higher chances of recovering from cardiac arrest than those exposed to prolonged CPR. Moreover, CPC outcomes in the patients who succumbed to medical issues revealed that 22% of the sample had good CPC outcomes and 78% recorded poor CPC outcomes. In harmony with the findings of Scott et al., good CPC outcomes resulted in faster recovery among cardiac arrest patients.

The AED is an excellent way of administering defibrillation to individuals facing out-of hospital cardiac arrest. Its application by conventional and unconventional first responders seems safe and efficacious [12]. Provisionary investigation of unrestricted access to defibrillation indicates that better survival outcomes after abrupt heart attacks are attainable. Besides, rhythm is also an important factor in cardiac arrest. We have four potential electrocardiographic rhythms. VF, VT, PEA, and asystole. VF is an unsystematic electrical action, whereas a VT produces systematic electrical pulses. Both VF and VT can provide adequate blood flow. PEA is a systematic rhythm typified by a lack of or inadequate mechanical ventricular action to give a noticeable pulse. Asystole is a lack of ventricular action in the presence or absence of atrial activity. Thus, health institutions must create a strategy to lower the interval between attack and quick defibrillation. Defibrillation results also improve if the intervals between chest compressions are minimal [13]. Findings from the research also demonstrated the relationship between the independent variables in this study and CPR outcomes. An influential factor in the recovery trend, age differences among subjects were translated in the results. According to the findings, youthfulness is associated with good CPC outcomes and a mean rank of 5.8 years, whereas a mean rank of 14.9 years was aligned with poor CPC outcomes among test subjects. This study also concluded that other underlying medical conditions limited recovery trends in older patients. This was in line with the findings of Goto et al. [14]. According to Cheema et al. [15], patients with numerous comorbidities have lower chances of recovering after CPR administration. In addition to age and underlying medical conditions, this study demonstrated a significant difference in recovery rates between male and female patients. Outcomes in patients who survived after CPR administration showed that 50% of the women who recovered had good CPC outcomes. However, 58.8% of the men with poor CPC outcomes survived, and only 41.2% of the men with good CPC outcomes survived. These results align with the findings of Morrison et al. [16], who reported significant differences in recovery trends between male and female patients. This study has also demonstrated such significant differences.

## Strengths and limitations

Cardiopulmonary resuscitation is an advanced medical intervention that recommends uninterrupted or continuous chest compressions among cardiac arrest patients in out-of-hospital settings. The research incorporates reliable statistical analysis and data collection methods derived from a specific hospital’s electronic medical database. However, demographic data from the patient’s health records exclude cardiac patients in hospital settings and failed to some information about quality of CPR, first heart rhythm, post-cardiac arrest care, and conducted a comparative analysis of alternative medical interventions.

The neurological outcomes were evaluated only at the time of hospital discharge. CPC scores after hospital discharge at 3 months or 6 months were not evaluated.

## Conclusion

This study indicates that shorter CPR durations lead to favorable neurological outcomes and survival among cardiac arrest patients. Survival among such patients is significantly affected by preexisting medical conditions [17]. However, favorable neurological outcomes and recovery rates were observed to be significantly influenced by intervention strategies adopted by clinicians. This study, therefore, recommends the adoption of intervention strategies that not only help cardiac arrest patients overcome underlying medical conditions but also bring about favorable neurological outcomes and survival. Moreover, this study concluded that patients’ demographic data, such as gender and age, significantly influence outcomes in cardiac arrest patients. This study recommends the adoption of differentiated intervention strategies for cardiac arrest patients to enhance clinicians’ capacities to address specific medical needs that may influence neurological outcomes in individual patients.

This study also suggests that extended CPR administration adversely affects elderly patients more. Longer CPR durations among such patients lower their chances of survival from cardiac arrest. Because CPR largely contributes to OHCA treatment and the return of spontaneous circulation, this study recommends a keen analysis of CPR duration by medical practitioners to avoid complications, particularly in elderly patients. Moreover, health organizations ought to teach CPR administration and the average beneficial duration to members of the public to inform them of the best out-of-hospital intervention strategies for individuals suffering from cardiac arrest. There is a need for further investigation into other factors. First, large sample size and multifactorial traits such as underlying health conditions were not considered in this research. It was assumed that OHCA patients had received advanced life support according to CPR guidelines. Second, the possibility of uncontrolled confounders cannot be ruled out because of the nature of the participants. For instance, data on various factors such as the location where the OHCA occurred and preexisting comorbidities as well as the quality of the first CPR administered were not considered in this study. It is evident that the quality of the first CPR administered has a substantial impact on the patient’s recovery. Integrated efforts among various authorities should be encouraged to establish a systemized strategy to raise cognizance and knowledge of cardiopulmonary resuscitation. This action would reduce preventable deaths by enhancing individuals' ability to respond effectively to the cases. Authorities must avail high-quality cardiopulmonary resuscitation training opportunities and integrate CPR resources into educational curricula. In addition, frequent practical training on the condition can help address it. Lastly, because most people regard the media as the primary source of information for most individuals who understand CPR, the relevant authorities must strive harder to use the medium more robustly to raise awareness. The channel can inform the public on how to perform the procedure, guide them to CPR-resource centers close to them, and hold general campaigns related to the issue.

## Data Availability

The datasets used and analysed during the current study are available from the corresponding author on reasonable request.

## Abbreviation

CPC: Cerebral performance category
